# Comparison of Anthropometric and Atherogenic Indices as Screening Tools of Metabolic Syndrome in the Kazakh Adult Population in Xinjiang

**DOI:** 10.3390/ijerph13040428

**Published:** 2016-04-16

**Authors:** Xiang-Hui Zhang, Mei Zhang, Jia He, Yi-Zhong Yan, Jiao-Long Ma, Kui Wang, Ru-Lin Ma, Heng Guo, La-Ti Mu, Yu-Song Ding, Jing-Yu Zhang, Jia-Ming Liu, Shu-Gang Li, Qiang Niu, Dong-Sheng Rui, Shu-Xia Guo

**Affiliations:** 1 Department of Public Health, Shihezi University School of Medicine, Shihezi 832000, China; 18999335467@163.com (X.-H.Z.); zmberry@foxmail.com (M.Z.); hejia123.shihezi@163.com (J.H.); erniu19880215@sina.com (Y.-Z.Y.); jiaojiaolong881202@163.com (J.-L.M.); kwang311@hotmail.com (K.W.); marulin@126.com (R.-L.M.); guoheng@shzu.edu.cn (H.G.); murat08123@163.com (L.-T.M.); 13399931625@163.com (Y.-S.D.); yfyxxzjy@126.com (J.-Y.Z.); liujiaming@shzu.edu.cn (J.-M.L.); lishugang@ymail.com (S.-G.L.); niuqiang1214@163.com (Q.N.); ruidongsheng@gmail.com (D.-S.R.); 2 Department of Pathology and Key Laboratory of Xinjiang Endemic and Ethnic Diseases, Ministry of Education, Shihezi University School of Medicine, Shihezi 832000, China

**Keywords:** metabolic syndrome, anthropometric indices, atherogenic indices, Kazakh, screening

## Abstract

**Objective:** To compare the screening ability of various anthropometric and atherogenic indices for Metabolic syndrome (MetS) using three common criteria and to evaluate the validity of suitable parameters in combination for the screening of MetS among a Kazakh population in Xinjiang. **Methods:** A total of 3752 individuals were selected using the stratified cluster random sampling method from nomadic Kazakhs (≥18 years old) in Xinyuan county, Xinjiang, China, which is approximately 4407 km away from the capital Beijing. MetS was defined by the International Diabetes Federation (IDF), National Cholesterol Education Program Adult Treatment Panel III (ATP III) and Joint Interim Statement (JIS) criteria. The receiver operating characteristic curve (ROC) was used to compare the area under the ROC curve (AUC) of each index. The sensitivity, specificity, Youden’s index and cut-offs of each index for the screening of MetS were calculated. **Results:** According to the IDF, ATP III and JIS criteria, 18.61%, 10.51%, and 24.83% of males and 23.25%, 14.88%, and 25.33% of females had MetS. According to the IDF criteria, the waist-to-height ratio (WHtR) was the index that most accurately identified individuals with and without MetS both in males (AUC = 0.872) and females (AUC = 0.804), with the optimal cut-offs of 0.53 and 0.52, respectively. According to both the ATP III and JIS criteria, the lipid accumulation product (LAP) was the best index to discriminate between individuals with and without MetS in males (AUC = 0.856 and 0.816, respectively) and females (AUC = 0.832 and 0.788, respectively), with optimal cut-offs of 41.21 and 34.76 in males and 28.16 and 26.49 in females, respectively. On the basis of the IDF standard, Youden’s indices of WHtR and LAP serial tests for the screening of MetS were 0.590 and 0.455 in males and females, respectively, and those of WHtR and LAP parallel tests were 0.608 and 0.479, accordingly. **Conclusion:** According to the IDF, ATP III and JIS criteria, both the WHtR and LAP were better indices for the screening of MetS. The WHtR and LAP parallel test was the most accurate.

## 1. Introduction

Metabolic syndrome (MetS) is a cluster of metabolic abnormalities, characterized as central obesity, dysglycemia, raised blood pressure, elevated triglyceride (TG) levels, and low high-density lipoprotein cholesterol (HDL-C) levels [1]. MetS is associated with cardiovascular disease, type 2 diabetes morbidity and mortality, and all-cause mortality [2]. It is alarming that the prevalence of MetS is high and increasing in both developing and developed nations [3,4,5,6]. Thus, early identification and treatment of individuals with MetS is essential to prevent the adverse consequences related to its development. However, the diagnostic criteria of MetS are complex to conduct, which makes early identification of individuals with MetS challenging. Recently, many studies have focused on anthropometric and atherogenic indices for the screening of MetS. Nevertheless, controversy still remains as to which index conveys the highest risk in different countries, ethnicities, and genders [7,8,9,10], and combined screening evaluation is limited.

Body mass index (BMI) has been widely used to measure obesity. However, the BMI does not reflect the body fat distribution, which is an important limitation, because there are reports that the metabolic complications of obesity are more closely associated with visceral adiposity than overall adiposity [11]. Thus, other parameters of obesity, such as waist-to-height ratio (WHtR) and waist-to-hip ratio (WHR) have been widely studied. Several studies have reported that WHtR is the best predictor for the detection of cardiometabolic risk factors, particularly in Asian populations [12,13,14,15], whereas WHR is the best anthropometric index for screening MetS [16,17,18]. Although BMI, WHtR and WHR are both simple and convenient anthropometric indices for epidemiological studies, their validity in evaluating obesity has been questioned because they are unable to differentiate between lean mass and fat mass [19]. Body Adiposity Index (BAI) is a composite index based on hip circumference and height. One study found that BAI was the most correlated variable with dual energy X-ray absorptiometry (DEXA)-derived percentage (%) body adiposity, and thus, it was proposed to be a better index of body adiposity [20]. Lipid accumulation product (LAP) is based on the combination of waist circumference and plasma triglyceride levels, and it was initially developed by Kahn as a novel index of central lipid accumulation to predict the risk of MetS [21]. In addition, a few studies have indicated that the triglyceride-to-high-density lipoprotein cholesterol ratio (TG/HDL-C) can best predict the presence of MetS [8,9,22].

Thus, further research is essential in population groups where the various anthropometric and atherogenic indices, including the recently proposed BAI and LAP, have not been generally analyzed and compared. Kazakh is a large nomadic nation in Xinjiang; they have lived in remote mountain pastures for generations, with little contact with the outside world. Their special ethnicity and living environment make them different from Chinese Hans and other ethnics. Our previous studies found that the prevalence of MetS in Kazakhs is higher than other nations in Xinjiang [23,24]. Thus, this study aimed to compare the screening ability of BMI, WHtR, WHR, BAI, LAP and TG/HDL-C, and to evaluate the validity of given parameters in single and combined screening tests of MetS in this population. Moreover, we aimed to provide basic data and theoretical evidence for the improvement of MetS criteria, and our findings may have an important public health implication for nomadic Kazakhs living in other countries such as Kazakhstan, Uzbekistan, and Russia.

## 2. Methods

### 2.1. Ethics Statement

This study was approved by the Institutional Ethics Review Board (IERB) of the First Affiliated Hospital of Shihezi University School of Medicine (IERB No. SHZ2010LL01). Standard university hospital guidelines, including informed consent, voluntary participation, confidentiality, and anonymity were followed. All of the participants provided their written informed consent prior to the start of the study.

### 2.2. Setting and Study Population

The survey was performed from 2009 to 2010 in Yili prefecture, which is approximately 4407 km (2739 miles) from Beijing, and approximately 98% of the population are minority Muslim Kazakhs. Participants were selected using the multistage (prefecture-county-township-village) stratified cluster random sampling method. First, we selected the representative prefecture (Yili) based on the geographical distributions of the minority populations in Xinjiang, a province in northwestern China. Second, we randomly selected one county in the prefecture and one township from the county (Nalati Township in Xinyuan County). Finally, a stratified sampling method was used to select the corresponding villages in each township (6 villages in Nalati Township). We interviewed local Kazakhs aged ≥18 years who had resided in the village for at least 6 months and successfully interviewed a total of 3752 individuals (mean age 43.97 years, 1494 males and 2258 females). The response rate was 87.1%. The age, gender and educational level of the participants were similar to the whole population in Kazakh in Xinjiang based on the Xinjiang statistical yearbook 2010 data.

### 2.3. Questionnaire Survey

Detailed information on each participant was collected based on a self-developed questionnaire in a face-to-face interview. The questionnaire included demographic information (*i.e.*, age, gender, ethnicity, education level, *etc.*) and personal lifestyles (*i.e.*, smoking, alcohol intake, physical activity, dietary habits, *etc.*) of the respondents.

### 2.4. Serial Test and Parallel Test

We define a serial test as a test in which the result was positive if and only if all screening tests were positive, and a parallel test as a test that the result was positive if any of the screening tests was positive. A serial test improved the specificity, but reduced the sensitivity while a parallel test did the opposite.

### 2.5. Anthropometric Measurements and Laboratory Tests

A physical examination, including measurements of height, weight, waist circumference, hip circumference, systolic and diastolic blood pressure, was performed. Blood samples were drawn from the cubital vein into tubes containing heparin sodium in the morning after an overnight fast. The detailed description of these methods has been published previously [25,26]. TG, HDL-C and fasting blood glucose (FBG) levels were measured using a biochemical auto-analyzer (Olympus AU 2700; Olympus Diagnostics, Hamburg, Germany) in the clinical laboratory at the First Affiliated Hospital of Shihezi University School of Medicine.

BMI was calculated as weight in kilograms divided by height in meters squared. WHtR was calculated by dividing the waist circumference by height. WHR was calculated as the ratio of the waist-to-hip circumferences. BAI was calculated using the following equation: BAI = ((hip circumference)/(height^1.5^) − 18) [20]. LAP was calculated as (waist circumference (cm) − 65) × TG (mmol/L) for males, and (waist circumference (cm) − 58) × TG (mmol/L) for females [21]. TG/HDL-C was calculated as the triglyceride concentration divided by high-density lipoprotein cholesterol concentration.

### 2.6. Definition of MetS

MetS was defined using the International Diabetes Federation (IDF) criteria [27], which included central obesity (waist circumference ≥90 cm in males or ≥80 cm in females, Chinese population waist circumference cutoffs), in addition to any 2 of the following 4 factors: Elevated TG level (>150 mg/dL or 1.69 mmol/L), reduced HDL-C (<40 mg/dL or 1.04 mmol/L in men, <50 mg/dL or 1.29 mmol/L in women), elevated systolic BP (≥130 mmHg) or diastolic BP (≥85 mmHg), and elevated fasting blood glucose (FBG) (≥100 mg/dL). For comparison with different criteria and other reported studies, the modified National Cholesterol Education Program Adult Treatment Panel III (ATP III) [28] and Joint Interim Statement (JIS) [29] were also applied to the study.

### 2.7. Statistical Analysis

We established a database using EpiData software (EpiData Association, Odense, Denmark) [30], and then checked data and logicality during 2011–2013. Data were analyzed using SPSS (Statistical Program for Social Sciences, version 17.0, 2008, Chicago, IL, USA) between 2014 and 2015. Continuous variables were presented as mean ± standard deviation (M ± SD) and were analyzed using the *t*-test. Categorical variables were expressed as numbers or percentages and were analyzed using the Chi-square test and trend test. The screening ability of various anthropometric and atherogenic indices to identify individuals with MetS was explored using receiver operating characteristic curve (ROC) analysis. Plots of sensitivity (true positives) *versus* 1 minus specificity (false positives) were constructed in both males and females for each of the parameters. The area under the curve (AUC) of the ROC and 95% confidence intervals (CIs) were used to determine which index showed the highest accuracy in screening MetS. The AUC is a measure of discrimination, and the AUC of 0.5, 0.6 ≤ AUC < 7, 7 ≤ AUC < 0.8, 0.8 ≤ AUC < 0.9, and ≥0.9 corresponded to no discrimination, poor, acceptable, excellent, and outstanding discrimination, respectively [31]. The optimal cut-off point of each of the parameters to identify individuals with MetS was defined by the maximum value of Youden’s index, which was calculated as sensitivity + specificity − 1. In addition, the validity of suitable parameters alone and in combination screening tests for MetS was assessed using the sensitivity, specificity, false negative rate, false positive rate and Youden’s index. All statistical tests were two-sided, and differences were considered statistically significant at *p*-values < 0.05.

## 3. Results

### 3.1. Characteristics of the Study Population

The participants with MetS (IDF criteria) tended to have significantly higher values for age, height, weight, WC, hip circumference, systolic BP, diastolic BP, TG, FPG, BMI, WHtR, WHR, BAI, LAP and TG/HDL-C and lower HDL-C level compared to subjects without MetS both in males and females (*p* < 0.01 for all). According to the IDF and ATP III criteria, the prevalence of MetS in females (23.25% and 14.88%, respectively) were significantly higher than that in males (18.61% and 10.51%, respectively), whereas according to the JIS criteria, there was no significant difference between males (24.83%) and females (25.33%) (*p* = 0.73) (Table 1).

### 3.2. AUC of Each Variable for the Screening of MetS Using ROC Analyses

**Figure 1:**
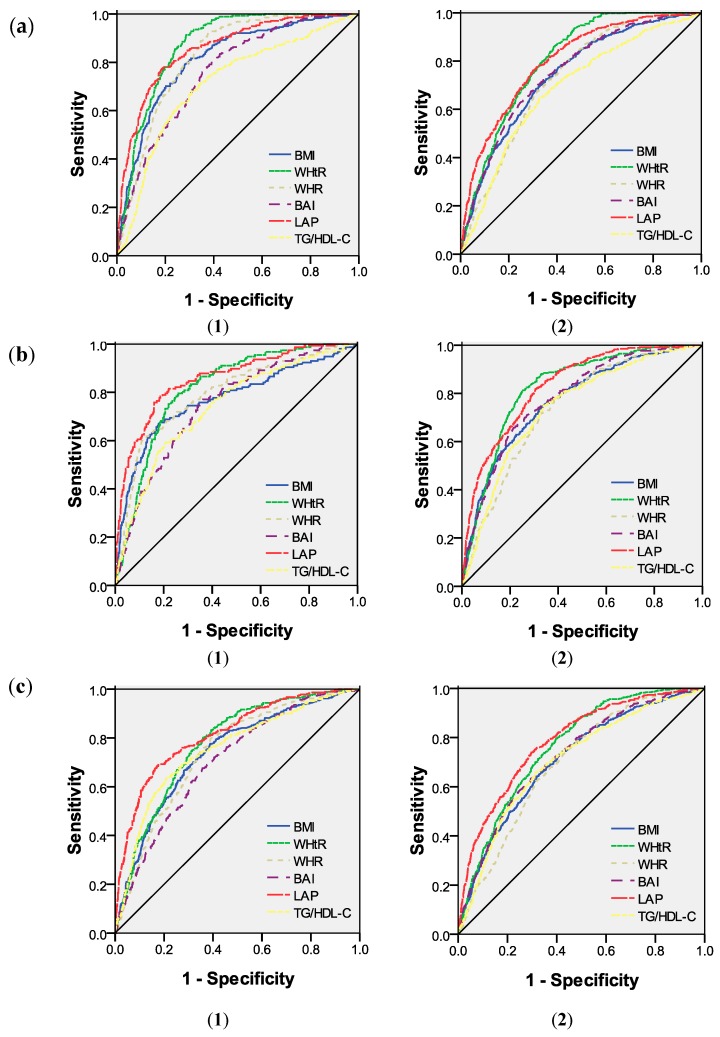
ROC curves for each variable for the screening of MetS in Kazakh males (**1**) and females (**2**) according to the: IDF (**a**); ATP III (**b**); and JIS (**c**) criteria.

According to the IDF criteria, in males, compared to the rest of the parameters, the WHtR had the highest AUC value (AUC = 0.872), followed by LAP, WHR, BMI, BAI and TG/HDL-C. In females, WHtR still had the highest AUC value (0.804), followed by LAP, BMI, BAI, WHR and TG/HDL-C. However, according to the ATP III and JIS criteria, LAP was best able to discriminate MetS in both males (AUC = 0.856 and 0.816, respectively) and females (AUC = 0.832 and 0.788, respectively), followed by WHtR (Figure 1 and Table 2).

### 3.3. Cut-of, Sensitivity, Specificity and Youden’s Index of Each Variable According to the Different Criteria

According to the IDF criteria, Youden’s index of WHtR was maximum compared to the remaining anthropometric and atherogenic indices in both males and females, with the optimal cut-off as 0.53 in males and 0.52 in females, followed by LAP, whereas TG/HDL-C had the minimum Youden’s index. According to the ATP III and JIS criteria, LAP had the maximum Youden’s index in both males and females, with the optimal cut-offs of 41.21 and 34.76, respectively, in males and 28.15 and 26.49, respectively, in females, followed by WHtR (Table 3).

### 3.4. Combined Screening Evaluation

The validity of WHtR and LAP in the combination screening of MetS in Kazakhs with WHtR ≥ 0.53 and LAP ≥ 34.76 for males and WHtR ≥ 0.52 and LAP ≥ 26.49 for females are shown in Table 4. On the basis of the IDF criteria, the WHtR and LAP parallel test had the maximum Youden’s index in both males and females, Youden’s index was 0.608 (Sen = 95.32%, Spe = 65.46%) in males and 0.479 (Sen = 93.90%, Spe = 54.01%) in females.

## 4. Discussion

A large cross-sectional study was performed during 2000–2001 by the InterASIA Study group. They reported that the prevalence of MetS in Chinese adults was 16.5% according to the IDF criteria and 13.7% according to the ATP III criteria, and the prevalence of MetS in females was significantly higher compared to males in nearly every age range [5]. Our study also found that the prevalence of MetS in Kazakhs was higher than the general level of Chinese subjects according to both the IDF criteria and ATP III criteria. After the data were stratified by gender, the prevalence of MetS in females was also significantly higher compared to males according to both the IDF criteria and ATP III criteria. Another study performed by Zuo *et al.* [32] reported that the age standardized prevalence of MetS in females (34.2%) was also higher than that in males (24.0%) according to the ATP III criteria. In addition, in recent years, studies in China and other countries have discovered the same conclusion [33,34,35]. However, when applying the JIS criteria, we found that 24.83% of males and 25.33% of females had MetS, and there was no significant difference between males and females. The main reason was that the cut-off of WC for central obesity was reduced from 90 cm (IDF and ATP III criteria) to 80 cm (JIS criteria), which resulted in an increased number of participants with central obesity and a significantly increased prevalence of MetS in males.

According to the IDF criteria, Zhang *et al.* [7] demonstrated that WHtR was the best index for the screening of MetS in the population of Guangdong province, China, with AUC values of 0.69 in males and 0.70 in females. In our study, the highest AUC values belonged to WHtR with values of 0.872 in Kazakh males and 0.804 in Kazakh females, which were much higher than those observed in Guangdong province. Taking height into account, WHtR may be a better predictor of metabolic risk and may more accurately reflect the MetS of Kazakhs in Xinjiang. One study has reported that shorter height was closely correlated with the risk of coronary heart disease [36]. Furthermore, Henriksson *et al.* [37] insisted that body height had a negative relationship to serum cholesterol and non-HDL-C in middle age males, and thus, it would be necessary to correct the WC for height.

According to both the ATP III and JIS criteria, when compared to the remaining various anthropometric and atherogenic indices, we found that LAP had the highest AUC in both males and females. The screening ability of WHtR for MetS demonstrated the second highest AUC in males and females. This result was consistent with the results obtained by Chiang *et al.* [10], who investigated 513 adults aged 50 and older in Taiwan. Similarly, another study using the ATP III criteria also obtained the same conclusion in Spanish adults [38]. Interestingly, we found the best index for the screening of MetS changed when applying different criteria. Therefore, we should select the proper parameter when based on different diagnostic criteria. LAP is a simple index, and it only requires the determination of serum TG and the measurement of WC. Moreover, the WC is not able to discriminate between visceral adipose tissue and subcutaneous adipose tissue, while visceral obesity is more closely associated with cardiometabolic risks compared to subcutaneous obesity [39]. Thus, when measuring obesity, it is important to use a routinely applicable index to estimate visceral adiposity. One study had reported that TG was significantly correlated to visceral adiposity in healthy males, even after controlling for abdominal subcutaneous adipose tissue [40]. Moreover, WC in combination with TG has been shown to be able to discriminate between individuals with the greatest amount of visceral adiposity [41] and those significantly associated with an increased risk of MetS [42].

Two studies on the Han population and Uighurs in Xinjiang were performed by Chen *et al.* [8,9]. They reported that TG/HDL-C was the best parameter for the screening of MetS in Han males and females according to the ATP III and JIS criteria, and in Uighur females according to the IDF criteria. However, the present study provided completely different results. When applying the IDF criteria, the AUC of TG/HDL-C was the lowest among the six indices. The potential reasons for such disagreement may be that the participants of these two studies aged 35 and older while those of ours were at age of 18 and older, and Kazakhs with MetS tended to have higher low HDL-C rates and lower high TG rates based on our previous study [23].

According to the IDF criteria, the optimal cut-off values of WHtR for the screening of MetS were 0.53 in Kazakh males and 0.52 in Kazakh females, which were much higher compared to those in Guangdong [7], Jiangsu [43], Japanese [44] and Korean [45] populations. Kazakh herdsmen keep a nomad diet with more horsemeat, milk, wheat, mutton, and dairy products as their primary foods. Dinner is the main meal, and there are almost no physical activities after dinner. High energy foods and lack of exercise may lead to the high cut-offs in Kazakhs. Genetic studies revealed that Kazakhs have a mixture of Mongolian and Caucasian backgrounds [25], which may explain the genetic influence on the studied Kazakhs, whose cut-off values are higher than that of other Asians. According to both the ATP III and JIS criteria, the optimal cut-off values of LAP for the screening of MetS were 41.21 and 34.76, respectively, in males and 28.15 and 26.49, respectively, in females. LAP is a novel atherogenic index for MetS, and relevant reports about this index in a Chinese population have been limited. In our study, the optimal cut-offs of LAP are different compared to other studies [10,46] due to the different diagnostic criteria, regions and countries. When using the IDF criteria as the gold standard, we found that the WHtR and LAP parallel test for the screening of MetS of Kazakhs had the highest sensitivity and Youden’s index in both males and females. However, the false positive rates for WHtR and LAP parallel tests were rather high, especially for females. The main reason may be due to sex difference in regional adipose tissue distribution and pattern of visceral fat deposition [47]. In fact, women had, on average, less visceral fat and more subcutaneous fat than men [48]. Therefore, women tended to be false positive in parallel tests. Furthermore, we found that the prevalence of MetS (IDF) was higher in females than that in males in this study. MetS is a cluster of metabolic abnormalities, and it can directly result in cardiovascular disease, type 2 diabetes and other adverse events. Early screening of MetS in a high risk population and dietary and lifestyle intervention play an important role in reducing the incidence of MetS and improving the quality of life. Thus, the WHtR and LAP parallel test may be much better tools compared to other screening tests.

The present study has several advantages. To the best of our knowledge, this is the first large-scale population-based study to compare the screening ability of various anthropometric and atherogenic indices for MetS according to different diagnostic criteria in Kazakhs. Furthermore, we demonstrated that both WHtR and LAP were better indices for the screening of MetS and provided the cut-offs of each index stratified according to sex. Further studies can use the cut-offs suggested in this study to screen MetS in a representative sample of Kazakh adults not only in China but worldwide. Furthermore, there are some limitations. First, it is a cross-sectional design, which precludes causal inferences. Second, the formula of LAP was derived from studies of white, non-Hispanic blacks and Mexican Americans, and included the minimum sex-specific WC values of 65 cm for males and 58 cm for females, respectively. Consistent with these criteria, individuals for whom the WC was less than this standard were not included in our study. The applicability of this formula to other countries, regions and ethnic groups has not been confirmed yet.

## 5. Conclusions

According to the IDF, ATP III and JIS criteria, the prevalence of MetS was 18.61%, 10.51%, and 24.83% in Kazakh males and 23.25%, 14.88%, and 25.33% in Kazakh females in Xinjiang, respectively. Among the six anthropometric and atherogenic indices, WHtR and LAP were better indices for the screening of MetS in both Kazakh males and Kazakh females according to different diagnostic criteria. Moreover, the WHtR and LAP parallel test had the best validity to identify individuals with and without MetS in the Kazakh population in Xinjiang.

## Figures and Tables

**Table 1:** Baseline characteristics of the study population according to the International Diabetes Federation (IDF) criteria.

Parameters	Male (*n* = 1494)		*p*	Female (*n* = 2258)		*p*
	With MetS (*n* = 278)	Without MetS (*n* = 1216)		With MetS (*n* = 525)	Without MetS (*n* = 1733)	
Age (years)	51.45 ± 11.09	43.51 ± 13.66	<0.001	50.34 ± 11.76	41.23 ± 12.55	<0.001
Height (cm)	171.43 ± 6.63	169.82 ± 7.04	<0.001	159.20 ± 6.67	158.14 ± 6.36	<0.001
Weight (kg)	80.83 ± 11.86	67.91 ± 10.77	<0.001	66.65 ± 11.14	58.32 ± 10.11	<0.001
WC (cm)	100.03 ± 7.78	84.91 ± 10.13	<0.001	92.51 ± 9.38	80.51 ± 10.85	<0.001
Hip circumference (cm)	105.39 ± 6.79	96.82 ± 7.55	<0.001	103.49 ± 8.50	95.30 ± 8.69	<0.001
Systolic BP (mmHg)	144.19 ± 21.54	128.52 ± 22.09	<0.001	140.47 ± 26.60	121.77 ± 21.41	<0.001
Diastolic BP (mmHg)	92.89 ± 14.15	82.31 ± 13.26	<0.001	90.48 ± 14.84	78.72 ± 13.33	<0.001
TG (mmol/L)	1.70 ± 0.78	1.16 ± 0.72	<0.001	1.31 ± 0.66	0.98 ± 0.49	<0.001
HDL-C (mmol/L)	1.21 ± 0.38	1.37 ± 0.56	<0.001	1.29 ± 0.55	1.49 ± 0.66	<0.001
FBG (mmol/L)	6.02 ± 1.08	4.89 ± 1.12	<0.001	5.55 ± 1.24	4.77 ± 0.92	<0.001
BMI	27.82 ± 3.73	23.53 ± 3.39	0.005	26.68 ± 4.27	23.13 ± 3.72	0.005
WHtR	0.59 ± 0.05	0.50 ± 0.05	<0.001	0.59 ± 0.06	0.51 ± 0.07	<0.001
WHR	0.95 ± 0.04	0.88 ± 0.06	<0.001	0.89 ± 0.05	0.84 ± 0.07	<0.001
BAI	29.48 ± 3.88	25.84 ± 3.85	<0.001	34.20 ± 5.11	29.73 ± 4.68	<0.001
LAP	59.43 ± 28.89	23.49 ± 19.28	<0.001	44.09 ± 22.12	22.13 ± 16.25	<0.001
TG/HDL-C	1.55 ± 0.88	1.02 ± 0.80	<0.001	1.17 ± 0.88	0.80 ± 0.69	<0.001
MetS-IDF	278 (18.61%)		-	525 (23.25%)		0.001
MetS-ATP III	157 (10.51%)		-	336 (14.88%)		<0.001
MetS-JIS	371 (24.83%)		-	572 (25.33%)		0.730

Note: Continuous variables presented as mean ± SD, sex differences explored using *t*-test. Categorical variables presented as *n* (%); and sex differences were explored using Chi-square test. WC, waist circumference; TG, triglycerides; HDL-C, high-density lipoprotein cholesterol; FBG, fasting blood glucose; BMI, body mass index; WHtR, waist-to-height ratio; WHR, waist-to-hip ratio; BAI, body adiposity index; LAP, lipid accumulation product.

**Table 2:** AUC of each variable for the screening of MetS in Kazakhs.

Parameters	IDF Criteria		ATP III Criteria		JIS Criteria	
	AUC (95% CI) in Male	AUC (95% CI) in Female	AUC (95% CI) in Male	AUC (95% CI) in Female	AUC (95% CI) in Male	AUC (95% CI) in Female
BMI	0.815 (0.788, 0.842)	0.744 (0.721, 0.767)	0.772 (0.727, 0.817)	0.771 (0.744, 0.798)	0.74 (0.712, 0.769)	0.713 (0.689, 0.737)
WHtR	**0.872 (0.854, 0.891)**	**0.804 (0.786, 0.822)**	0.818 (0.787, 0.848)	0.825 (0.803, 0.847)	0.777 (0.751, 0.802)	0.764 (0.743, 0.784)
WHR	0.827 (0.804, 0.849)	0.731 (0.709, 0.753)	0.795 (0.756, 0.834)	0.74 (0.714, 0.766)	0.741 (0.713, 0.768)	0.698 (0.675, 0.721)
BAI	0.761 (0.733, 0.790)	0.754 (0.731, 0.776)	0.747 (0.709, 0.786)	0.783 (0.758, 0.808)	0.705 (0.675, 0.734)	0.726 (0.702, 0.749)
LAP	0.858 (0.834, 0.882)	0.801 (0.781, 0.822)	**0.856 (0.823, 0.889)**	**0.832 (0.811, 0.854)**	**0.816 (0.791, 0.842)**	**0.788 (0.767, 0.809)**
TG/HDL-C	0.715 (0.681, 0.749)	0.688 (0.663, 0.714)	0.739 (0.697, 0.780)	0.742 (0.715, 0.770)	0.752 (0.723, 0.782)	0.707 (0.683, 0.732)

Note: Variables with highest AUC value in **bold**.

**Table 3:** The cut-off, sensitivity, specificity and Youden’s index of each variable for the screening of MetS in Kazakh males and females.

Parameters	IDF Criteria				ATP III Criteria				JIS Criteria			
	Cut-off	Sen (%)	Spe (%)	Youden’s Index	Cut-off	Sen (%)	Spe (%)	Youden’s Index	Cut-off	Sen (%)	Spe (%)	Youden’s Index
Male												
BMI	24.84	79.10	72.00	0.511	26.72	67.50	81.70	0.492	23.73	77.60	61.10	0.387
WHtR	0.53	88.49	72.04	**0.605**	0.58	79.60	74.00	0.536	0.52	80.50	64.10	0.446
WHR	0.89	90.40	62.20	0.526	0.94	61.10	88.40	0.495	0.89	80.30	60.20	0.405
BAI	26.82	77.70	64.10	0.418	27.14	77.10	63.80	0.409	26.82	67.70	64.30	0.32
LAP	34.76	77.70	81.10	0.588	41.21	78.30	81.40	**0.597**	34.76	68.50	82.90	**0.514**
TG/HDL-C	1.08	68.30	68.80	0.371	1.20	59.20	79.30	0.385	1.20	63.90	78.60	0.425
Female												
BMI	24.35	68.40	68.70	0.371	24.50	73.80	67.40	0.412	24.33	64.20	68.10	0.323
WHtR	0.52	84.00	62.44	**0.464**	0.55	81.80	69.50	0.513	0.52	79.40	60.40	0.398
WHR	0.85	78.90	57.20	0.361	0.86	77.40	61.70	0.391	0.85	74.30	56.70	0.31
BAI	31.45	69.10	68.00	0.371	33.16	66.40	77.80	0.442	31.45	65.00	68.70	0.337
LAP	26.49	76.20	69.80	0.460	28.15	81.30	74.60	**0.559**	26.49	74.00	70.30	**0.443**
TG/HDL-C	0.79	64.60	67.40	0.320	0.79	75.00	66.10	0.411	0.79	65.70	68.70	0.344

Note: Variables with highest Youden’s index in **bold**. Sen, sensitivity; Spe, specificity.

**Table 4:** The validity of WHtR, LAPin combination for the screening of MetS in a Kazakh population.

Screening	Sen (%)	Spe (%)	False Negative Rate (%)	False Positive Rate (%)	Youden’s Index
Male					
WHtR, LAP parallel test	95.32	65.46	4.68	34.54	**0.608**
WHtR, LAP serial test	70.86	88.16	29.14	11.84	0.590
Female					
WHtR, LAP parallel test	93.90	54.01	6.10	45.99	**0.479**
WHtR LAP serial test	66.29	78.19	33.71	21.81	0.445

Note: variables with highest Youden’s index in **bold**. Sen, sensitivity, Spe, specificity.
